# Supplementary material for: Enhanced Nitrous Oxide Production in Denitrifying Dechloromonas aromatica Strain RCB Under Salt or Alkaline Stress Conditions
Source: Front Microbiol. 2019 Jun 5;10:1203. doi: 10.3389/fmicb.2019.01203 (PMC6593283; doi:10.3389/fmicb.2019.01203)
Supplement: Supplementary file 1 [file Data_Sheet_1.docx]

**Supplementary Material**

**Enhanced nitrous oxide production in denitrifying *Dechloromonas aromatica* strain RCB under salt or alkaline stress conditions**

Heejoo Han^1^, Bongkeun Song^1,2^, Min Joon Song,^1^ and Sukhwan Yoon^1*^

^1^Department of Civil and Envionmental Engineering, Korea Advanced Institute of Science and Technology, Daejeon, 350-701, Korea

^2^Department of Biological Sciences, Virginia Institute of Marine Sciences, College of William and Mary, Gloucester Point, VA, USA

**Fig. S1** Reduction of 5 mM NO_3_^-^ by the denitrifying *Dechloromonas aromatica* RCB cultures incubated at varying NaCl concentration and pH: **(A)** the control condition (0.05% w/v NaCl, pH 7.0); **(B)** 0.3% w/v NaCl, pH 7.0; **(C)** 0.5% w/v NaCl, pH 7.0; **(D)** 0.7% w/v NaCl, pH 7.0; **(E)** 0.9% w/v NaCl, pH 7.0; **(F)** 0.05% w/v NaCl, pH 7.5; and **(G)** 0.05% w/v NaCl, pH 8.0. The amounts of N_2_O-N (■) and the absorbances at 600 nm (●) were monitored until the stationary phase was reached. The data points represent the averages of triplicate cultures and the error bars the standard deviations of the triplicate measurements.

**Fig. S2** The growth curves of aerobic *Dechloromonas aromatica* RCB cultures incubated at varying NaCl concentration and pH: **(A)** the control condition (0.05% w/v NaCl, pH 7.0); **(B)** 0.3% w/v NaCl, pH 7.0; **(C)** 0.5% w/v NaCl, pH 7.0; **(D)** 0.7% w/v NaCl, pH 7.0; **(E)** 0.9% w/v NaCl, pH 7.0; **(F)** 0.05% w/v NaCl, pH 7.5; and **(G)** 0.05% w/v NaCl, pH 8.0. The absorbances at 600 nm (●) were monitored until the nitrogen oxides were depleted or upon confirmation of the termination of denitrification reaction. The data points represent the averages of triplicate cultures and the error bars the standard deviations of the triplicate measurements.

**Fig. S3.** Reduction of 5 mM NO_3_^-^ by the denitrifying *Dechloromonas aromatica* RCB cultures incubated at the alkaline stress condition (0.05% w/v NaCl, pH 8.0) amended with 10% v/v C_2_H_2_ in the headspace. The amounts of NO_3_^-^ (●), NO_2_^-^ (●), and N_2_O-N (●) and the absorbances at 600 nm (●) were monitored until the termination of denitrification reaction. Each reaction vessel is a 160 mL serum bottle with the initial aqueous phase volume of 100 mL. The data points represent the averages of triplicate cultures and the error bars the standard deviations of the triplicate measurements.

**Fig. S4.** The values of *nosZ*/*norB* (▲) calculated with the *norB*, and *nosZ* transcription data obtained from incubation of *D. aromatica* strain RCB at **(A)** the control condition (0.05% w/v NaCl, pH 7.0), **(B)** the salt stress condition (0.7% w/v NaCl) and the **(C)** pH stress condition (pH 8.0). The errors were calculated using the error propagation method computed with the standard deviations of triplicate biological replicates. The amounts of N_2_O (■) in the reaction vessels are included for convenient comparison between the datasets.

**Fig. S1**


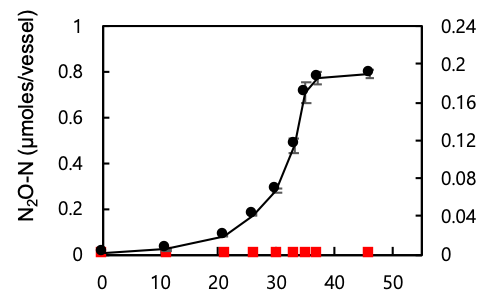

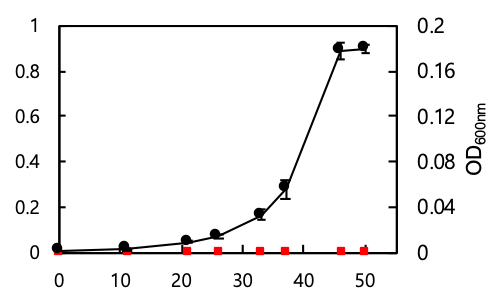

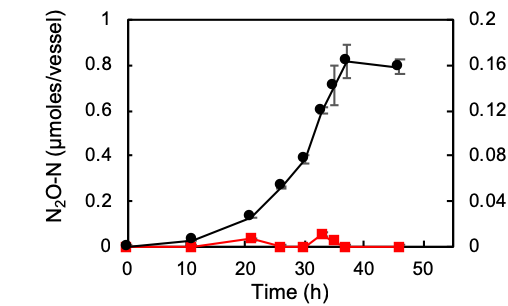

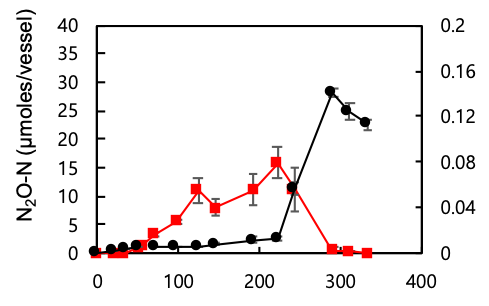

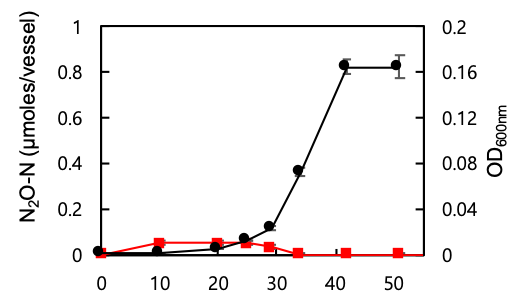

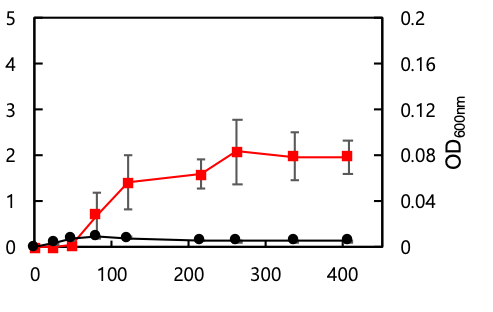

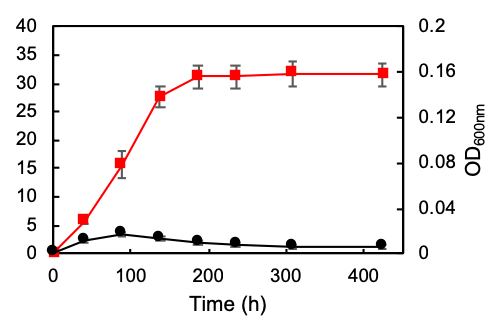


**(A)**

**(E)**

**(F)**

**(B)**

**(C)**

**(D)**

**(G)**

**Fig. S2**


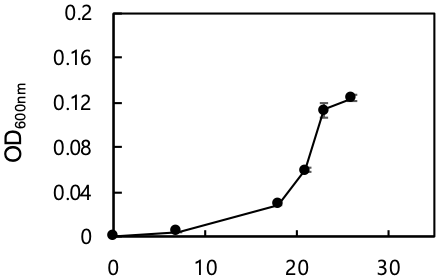

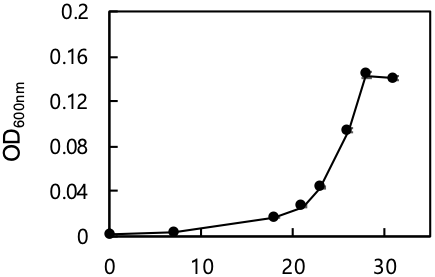

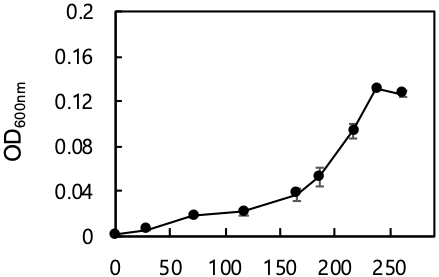


**(A)**

**(B)**

**(C)**

**(F)**

**(D)**


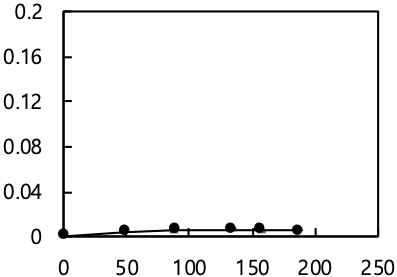


**(E)**

**(G)**


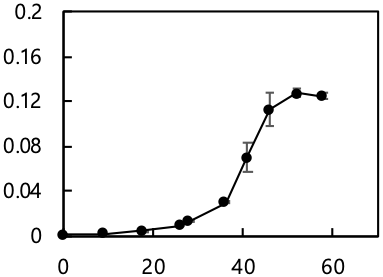

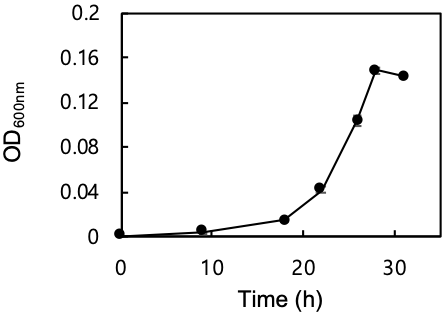

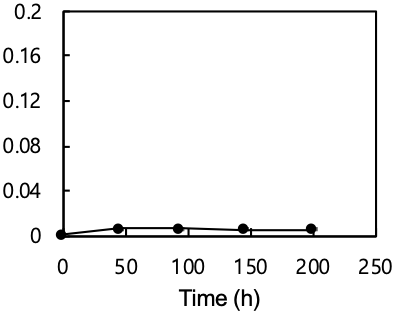


**Fig. S3**

**Fig. S4**


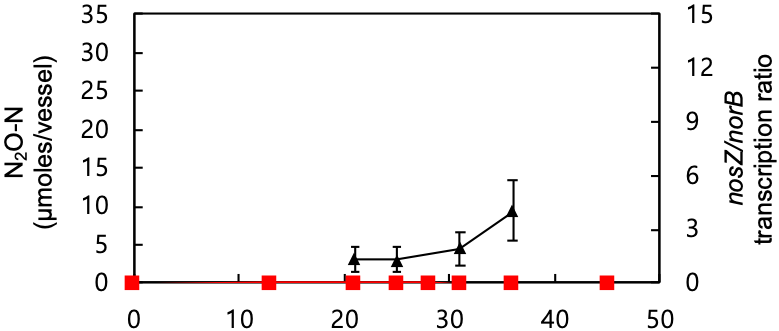

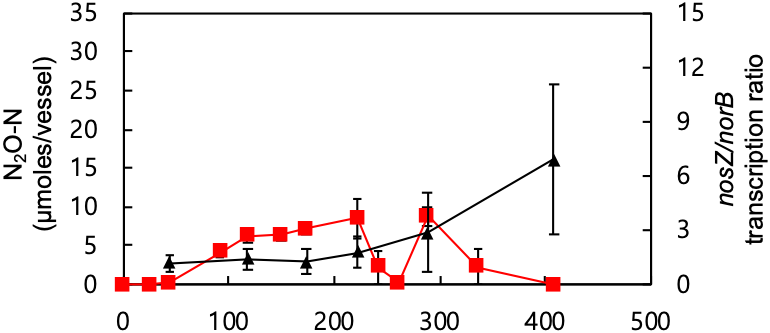

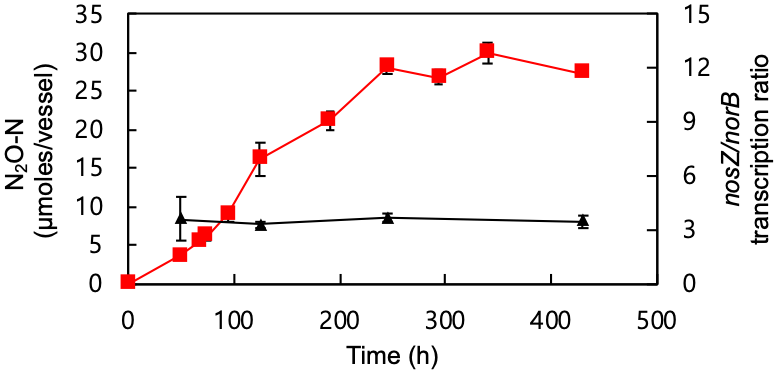


**(A)**

**(B)**

**(C)**

**Table. S1.** The primer sets used for the RT-qPCR assays

| **Primer** | **Sequence (5’→3’)** | **Target**  **gene** | **Amplicon**  **Length (bp)** | **Slope** | **y-intercept** | **Amplification efficiency** | **R^2^** | **Reference** |
| --- | --- | --- | --- | --- | --- | --- | --- | --- |
| *nirS1489f* | CTACCGGCAAGAACCTTGAG | *nirS_1_*  (Daro_3274) | 210 | -3.321 | 34.867 | 100.0 | 0.998 | This study |
| *nirS1678r* | AAACGTAGTGGGGATGCTTG |  |  |  |  |  |  |  |
| *nirS902f* | GTACCGGCAATCGCATACTT | *nirS_2_*  (Daro_3323) | 237 | -3.324 | 33.070 | 99.9 | 1.0 | This study |
| *nirS1138r* | CGGTGACAGCAAGAAGATCA |  |  |  |  |  |  |  |
| *nosZ799f* | GGGCATGTCGTACAGGAGTT | *nosZ*  (Daro_1575) | 239 | -3.320 | 35.659 | 100.1 | 0.999 | This study |
| *nosZ1037r* | CCCAAGTCGTCAAGTGGAAT |  |  |  |  |  |  |  |
| *norB463f* | GTGCTGAAGGGCAAGAAGAC | *norB*  (Daro_3191) | 247 | -3.371 | 34.124 | 98.0 | 1.0 | This study |
| *norB709r* | TGACGTACAGCCACTTTTCG |  |  |  |  |  |  |  |
| *recA831f* | CCGGACCATAGATTTCAACG | *recA*  (Daro_4152) | 170 | -3.446 | 35.010 | 95.1 | 0.999 | This study |
| *recA999r* | GCACTGCAACAGATCGAAAA |  |  |  |  |  |  |  |
| *luc_refA* | TACAACACCCCAACATCTTCGA | luciferase  control  cDNA | 67 | -3.330 | 35.417 | 99.7 | 0.999 | Johnson  et al.,  2005 |
| *luc_refB* | GGAAGTTCACCGGCGTCAT |  |  |  |  |  |  |  |

**Reference**

Johnson DR, Lee PK, Holmes VF, Alvarez-Cohen L. (2005). An internal reference technique for accurately quantifying specific mRNAs by real-time PCR with application to the *tceA* reductive dehalogenase gene. *Appl Environ Microbiol,* 71. 3866-3871. doi: 10.1128/AEM.71.7.3866-3871.2005
